# Poly[[triaqua­(μ_3_-pyridine-2,4,6-tricarboxyl­ato)terbium(III)] monohydrate]

**DOI:** 10.1107/S1600536812028929

**Published:** 2012-06-30

**Authors:** Hong-lin Zhu

**Affiliations:** a Crystal Engineering Division, Center of Applied Solid State Chemistry Research, Ningbo University, Ningbo, Zhejiang 315211, People’s Republic of China

## Abstract

The asymmetric unit of the title compound, {[Tb(C_8_H_2_NO_6_)(H_2_O)_3_]·H_2_O}_*n*_, contains one Tb^III^ ion, one pyridine-2,4,6-tricarboxyl­ate (ptc) anion, three aqua ligands and one lattice water mol­ecule. The Tb^III^ ion is nine coordinated by one N and five O atoms from three ptc ligands and by three O atoms from the three aqua ligands in a distorted bicapped trigonal–prismatic geometry. The ptc ligands bridge the Tb^III^ ions into a two-dimensional polymeric framework parallel to (100). An extensive O—H⋯O hydrogen-bonding network consolidates the crystal packing.

## Related literature

For the crystal structures of related complexes, see: Das *et al.* (2009 ▶); Wang & Zhang (2009 ▶); Wang *et al.* (2010 ▶); Lin *et al.* (2011 ▶); Jin *et al.* (2012 ▶).
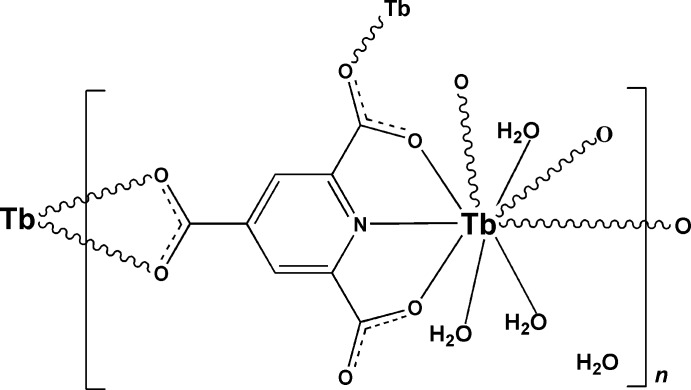


## Experimental

### 

#### Crystal data

[Tb(C_8_H_2_NO_6_)(H_2_O)_3_]·H_2_O
*M* *_r_* = 439.09
Monoclinic, 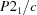

*a* = 11.936 (2) Å
*b* = 7.3343 (15) Å
*c* = 13.516 (3) Å
β = 96.43 (3)°
*V* = 1175.8 (4) Å^3^
*Z* = 4
Mo *K*α radiation
μ = 6.07 mm^−1^
*T* = 293 K
0.32 × 0.30 × 0.28 mm

#### Data collection

Rigaku R-AXIS RAPID diffractometer
Absorption correction: multi-scan (*ABSCOR*; Higashi, 1995 ▶) *T*_min_ = 0.164, *T*_max_ = 0.183
10998 measured reflections
2664 independent reflections
2528 reflections with *I* > 2σ(*I*)
*R*_int_ = 0.066

#### Refinement

*R*[*F* ^2^ > 2σ(*F* ^2^)] = 0.028
*wR*(*F* ^2^) = 0.067
*S* = 1.06
2664 reflections
182 parameters
H-atom parameters constrained
Δρ_max_ = 2.18 e Å^−3^
Δρ_min_ = −1.11 e Å^−3^

### 

Data collection: *RAPID-AUTO* (Rigaku, 1998 ▶); cell refinement: *RAPID-AUTO*; data reduction: *CrystalStructure* (Rigaku/MSC, 2004 ▶); program(s) used to solve structure: *SHELXS97* (Sheldrick, 2008 ▶); program(s) used to refine structure: *SHELXL97* (Sheldrick, 2008 ▶); molecular graphics: *SHELXTL* (Sheldrick, 2008 ▶); software used to prepare material for publication: *SHELXL97*.

## Supplementary Material

Crystal structure: contains datablock(s) global, I. DOI: 10.1107/S1600536812028929/cv5315sup1.cif

Structure factors: contains datablock(s) I. DOI: 10.1107/S1600536812028929/cv5315Isup2.hkl

Additional supplementary materials:  crystallographic information; 3D view; checkCIF report

## Figures and Tables

**Table 1:** Hydrogen-bond geometry (Å, °)

*D*—H⋯*A*	*D*—H	H⋯*A*	*D*⋯*A*	*D*—H⋯*A*
O7—H7*A*⋯O5^i^	0.83	1.98	2.642 (4)	136
O7—H7*B*⋯O4^ii^	0.84	2.45	2.966 (4)	120
O8—H8*A*⋯O5^i^	0.85	2.04	2.822 (4)	152
O8—H8*B*⋯O10^iii^	0.81	1.95	2.760 (5)	174
O9—H9*A*⋯O1^iv^	0.81	2.00	2.808 (4)	179
O9—H9*B*⋯O3^v^	0.81	2.00	2.805 (4)	179
O10—H10*A*⋯O6^vi^	0.84	2.33	2.977 (5)	134
O10—H10*B*⋯O7^vi^	0.85	2.55	3.169 (5)	130

Symmetry codes: (i) 
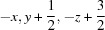
; (ii) 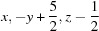; (iii) 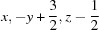; (iv) 
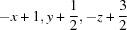
; (v) 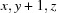; (vi) 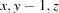.

**Fig. 1.**
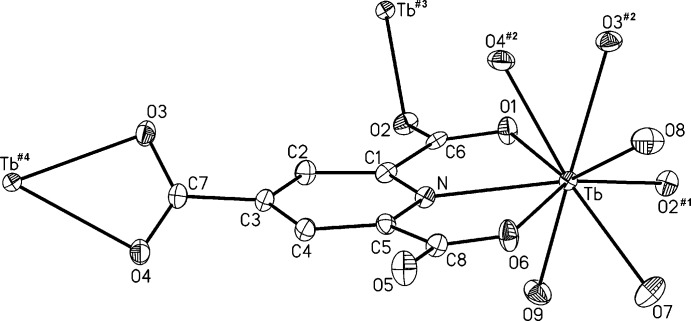
A portion of the crystal structure of (I) showing the atomic numbering and 45% probability dispalcement ellipsoids [symmetry codes: (#1) -x + 1, y + 1/2, -z + 3/2; (#2) x, -y + 3/2, z - 1/2; (#3) -x + 1, y - 1/2, -z + 3/2; (#4) x, -y + 3/2, z + 1/2]. H atoms omitted for clarity.

**Fig. 2.**
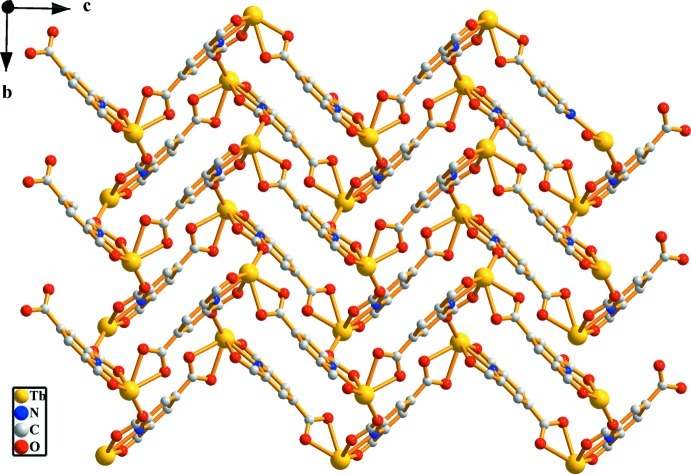
A portion of the two-dimensional polymeric framework in (I) viewed down the a axis. H atoms omitted for clarity

## supplementary crystallographic information

### Comment

As an ongoing part of our investigations of the lanthanide complexes with pyridine-2,4,6-tricarboxylate (Jin *et al.*, 2012; Lin *et al.*, 2011), we report here the title compound with Tb (I).

In (I) (Fig.1), all bond lengths and angles are normal and correspond to those observed in isostructural polymeric compounds with Gd (Wang & Zhang, 2009), Sm (Wang *et al.*, 2010) and Dy (Wang *et al.*, 2010; Das *et al.*, 2009). Each Tb center is coordinated by three aqua ligands and three ptc ligands (H_3_ptc = pyridine-2,4,6-tricarboxylate) to form 4,4'-bicapped trigonal prismatic TbNO_8_ chromophore with a 4,4'-bicapped trigonal prismatic environment. The pyridyl N atom, 2-position and 6-position carboxylate group coordinated two Tb atoms, and the 4-position carboxylate group chelated one Tb atom.

The TbNO_8_ chromophores are bridged by the ptc anions to form two-dimensional corrugated herringbone-like layers, which extend infinitely parallel to (100) (Fig. 2). The aqua ligands (O7, O8 and O9) donate hydrogen atom to carboxylate oxygen atoms (O4, O3 and O1) to form intralayer hydrogen bonds, and simultaneously O8 and O9 donate hydrogen atom to the carboxylate O5 to form interlayer hydrogen bonds (Table 1). Obviously, the former intralayer hydrogen bonding interactions contribute to stabilization of the two-dimensional layer, and the latter are found to be responsible for supramolecular assembly of the two-dimensional layers into a three-dimensional supramolecular architecture.

### Experimental

A mixture of Tb_4_O_7_ (0.0556 g, 0.075 mmol), pyridine-2,4,6-tricarboxylic acid (0.0649 g, 0.3 mmol) and H_2_O (10 ml) was sealed into a 23 ml Teflon-lined stainless autoclave, which was heated up to 180°C for 4 days, and then cooled to room temperature. A small amount of colourless needle-shaped crystals were obtained.

### Refinement

H atoms bonded to C atoms were palced in geometrically calculated position and were refined using a riding model, with *U*_iso_(H) = 1.2 *U*_eq_(C). H atoms attached to O atoms were found in a difference Fourier synthesis and were refined using a riding model, with the O–H distances fixed as initially found and with *U*_iso_(H) values set at 1.2-1.5 *U*eq(O).

### Crystal data

**Table:**

[Tb(C_8_H_2_NO_6_)(H_2_O)_3_]·H_2_O	*F*(000) = 840
*M**_r_* = 439.09	*D*_x_ = 2.480 Mg m^−^^3^
Monoclinic, *P*2_1_/*c*	Mo *K*α radiation, λ = 0.71073 Å
Hall symbol: -P 2ybc	Cell parameters from 9883 reflections
*a* = 11.936 (2) Å	θ = 3.0–27.4°
*b* = 7.3343 (15) Å	µ = 6.07 mm^−^^1^
*c* = 13.516 (3) Å	*T* = 293 K
β = 96.43 (3)°	Block, colorless
*V* = 1175.8 (4) Å^3^	0.32 × 0.30 × 0.28 mm
*Z* = 4	

### Data collection

**Table:**

Rigaku R-AXIS RAPID diffractometer	2664 independent reflections
Radiation source: fine-focus sealed tube	2528 reflections with *I* > 2σ(*I*)
Graphite monochromator	*R*_int_ = 0.066
Detector resolution: 0 pixels mm^-1^	θ_max_ = 27.4°, θ_min_ = 3.0°
ω scan	*h* = −15→15
Absorption correction: multi-scan (*ABSCOR*; Higashi, 1995)	*k* = −9→9
*T*_min_ = 0.164, *T*_max_ = 0.183	*l* = −17→15
10998 measured reflections	

### Refinement

**Table:**

Refinement on *F*^2^	Secondary atom site location: difference Fourier map
Least-squares matrix: full	Hydrogen site location: inferred from neighbouring sites
*R*[*F*^2^ > 2σ(*F*^2^)] = 0.028	H-atom parameters constrained
*wR*(*F*^2^) = 0.067	*w* = 1/[σ^2^(*F*_o_^2^) + (0.0172*P*)^2^ + 1.9544*P*] where *P* = (*F*_o_^2^ + 2*F*_c_^2^)/3
*S* = 1.06	(Δ/σ)_max_ = 0.001
2664 reflections	Δρ_max_ = 2.18 e Å^−^^3^
182 parameters	Δρ_min_ = −1.11 e Å^−^^3^
0 restraints	Extinction correction: *SHELXL97* (Sheldrick, 2008), Fc^*^=kFc[1+0.001xFc^2^λ^3^/sin(2θ)]^-1/4^
Primary atom site location: structure-invariant direct methods	Extinction coefficient: 0.0031 (4)

### Special details

**Table:**

Geometry. All e.s.d.'s (except the e.s.d. in the dihedral angle between two l.s. planes) are estimated using the full covariance matrix. The cell e.s.d.'s are taken into account individually in the estimation of e.s.d.'s in distances, angles and torsion angles; correlations between e.s.d.'s in cell parameters are only used when they are defined by crystal symmetry. An approximate (isotropic) treatment of cell e.s.d.'s is used for estimating e.s.d.'s involving l.s. planes.
Refinement. Refinement of *F*^2^ against ALL reflections. The weighted *R*-factor *wR* and goodness of fit *S* are based on *F*^2^, conventional *R*-factors *R* are based on *F*, with *F* set to zero for negative *F*^2^. The threshold expression of *F*^2^ > σ(*F*^2^) is used only for calculating *R*-factors(gt) *etc*. and is not relevant to the choice of reflections for refinement. *R*-factors based on *F*^2^ are statistically about twice as large as those based on *F*, and *R*- factors based on ALL data will be even larger.

### Fractional atomic coordinates and isotropic or equivalent isotropic displacement parameters (Å^2^)

**Table:**

	*x*	*y*	*z*	*U*_iso_*/*U*_eq_	
Tb	0.285028 (12)	1.21386 (2)	0.704284 (11)	0.01088 (9)	
N	0.2959 (2)	1.0044 (4)	0.8499 (2)	0.0132 (5)	
C1	0.3926 (3)	0.9190 (5)	0.8812 (2)	0.0140 (6)	
C2	0.3992 (3)	0.7853 (5)	0.9547 (3)	0.0165 (7)	
H2A	0.4669	0.7266	0.9750	0.020*	
C3	0.3023 (3)	0.7424 (5)	0.9978 (3)	0.0167 (7)	
C4	0.2025 (3)	0.8380 (5)	0.9689 (3)	0.0160 (7)	
H4A	0.1382	0.8183	1.0000	0.019*	
C5	0.2033 (3)	0.9641 (5)	0.8917 (2)	0.0141 (7)	
C6	0.4904 (3)	0.9703 (4)	0.8255 (2)	0.0137 (6)	
O1	0.4681 (2)	1.0675 (4)	0.74843 (18)	0.0186 (5)	
O2	0.58643 (19)	0.9109 (3)	0.85646 (18)	0.0168 (5)	
C7	0.2986 (3)	0.5894 (5)	1.0710 (2)	0.0162 (7)	
O3	0.3445 (2)	0.4402 (4)	1.05122 (18)	0.0209 (5)	
O4	0.2479 (2)	0.6092 (4)	1.14653 (19)	0.0238 (6)	
C8	0.0984 (3)	1.0619 (5)	0.8451 (3)	0.0163 (7)	
O5	0.0075 (2)	1.0267 (4)	0.8774 (2)	0.0250 (6)	
O6	0.1127 (2)	1.1688 (4)	0.7744 (2)	0.0228 (6)	
O7	0.1990 (2)	1.5188 (4)	0.7163 (2)	0.0262 (6)	
H7A	0.1317	1.5476	0.7166	0.031*	
H7B	0.2563	1.5872	0.7231	0.031*	
O8	0.1356 (3)	1.2471 (4)	0.5676 (2)	0.0296 (7)	
H8A	0.0844	1.3275	0.5632	0.050*	
H8B	0.1167	1.1746	0.5237	0.050*	
O9	0.3781 (2)	1.3706 (4)	0.85261 (19)	0.0243 (6)	
H9A	0.4225	1.4272	0.8240	0.029*	
H9B	0.3690	1.3909	0.9095	0.029*	
O10	0.0703 (4)	0.4780 (5)	0.9079 (3)	0.0481 (9)	
H10A	0.0413	0.4022	0.8662	0.058*	
H10B	0.1358	0.4736	0.8891	0.058*	

### Atomic displacement parameters (Å^2^)

**Table:**

	*U*^11^	*U*^22^	*U*^33^	*U*^12^	*U*^13^	*U*^23^
Tb	0.00838 (12)	0.01147 (13)	0.01279 (13)	−0.00008 (5)	0.00110 (8)	0.00033 (5)
N	0.0100 (13)	0.0133 (13)	0.0162 (13)	−0.0010 (11)	0.0007 (11)	0.0008 (11)
C1	0.0118 (15)	0.0129 (16)	0.0164 (15)	0.0003 (12)	−0.0022 (13)	−0.0011 (12)
C2	0.0119 (17)	0.0182 (19)	0.0188 (18)	0.0026 (12)	−0.0008 (15)	0.0034 (12)
C3	0.0205 (18)	0.0126 (15)	0.0168 (18)	0.0009 (14)	0.0015 (16)	0.0023 (13)
C4	0.0133 (16)	0.0159 (17)	0.0200 (17)	0.0012 (13)	0.0073 (14)	0.0029 (14)
C5	0.0141 (15)	0.0150 (17)	0.0127 (15)	0.0000 (12)	0.0000 (13)	0.0005 (12)
C6	0.0140 (15)	0.0084 (15)	0.0185 (16)	0.0013 (12)	0.0015 (13)	−0.0025 (12)
O1	0.0135 (11)	0.0220 (13)	0.0209 (12)	0.0040 (10)	0.0048 (10)	0.0084 (10)
O2	0.0109 (11)	0.0172 (13)	0.0215 (12)	0.0043 (9)	−0.0015 (10)	−0.0011 (10)
C7	0.0151 (16)	0.0159 (17)	0.0173 (16)	−0.0001 (13)	0.0001 (14)	0.0042 (13)
O3	0.0251 (13)	0.0172 (13)	0.0209 (12)	0.0047 (10)	0.0049 (11)	0.0059 (10)
O4	0.0320 (15)	0.0197 (14)	0.0214 (13)	0.0071 (11)	0.0108 (12)	0.0069 (10)
C8	0.0132 (15)	0.0160 (16)	0.0200 (16)	0.0008 (13)	0.0027 (14)	0.0017 (13)
O5	0.0092 (11)	0.0335 (16)	0.0321 (14)	−0.0009 (10)	0.0022 (11)	0.0106 (12)
O6	0.0162 (12)	0.0288 (15)	0.0242 (14)	0.0063 (11)	0.0052 (11)	0.0133 (11)
O7	0.0150 (12)	0.0184 (14)	0.0441 (17)	0.0012 (10)	−0.0016 (12)	0.0001 (12)
O8	0.0257 (16)	0.0348 (16)	0.0257 (16)	0.0121 (12)	−0.0084 (14)	−0.0057 (12)
O9	0.0280 (14)	0.0269 (15)	0.0180 (12)	−0.0104 (12)	0.0029 (11)	−0.0044 (11)
O10	0.067 (2)	0.041 (2)	0.0368 (17)	0.0117 (18)	0.0046 (18)	−0.0032 (15)

### Geometric parameters (Å, º)

**Table:**

Tb—O2^i^	2.324 (2)	C5—C8	1.518 (4)
Tb—O6	2.382 (3)	C6—O2	1.253 (4)
Tb—O8	2.432 (3)	C6—O1	1.266 (4)
Tb—O1	2.448 (2)	O2—Tb^iii^	2.324 (2)
Tb—O9	2.465 (3)	C7—O4	1.253 (4)
Tb—O7	2.474 (3)	C7—O3	1.266 (4)
Tb—N	2.488 (3)	C7—Tb^iv^	2.878 (3)
Tb—O4^ii^	2.518 (3)	O3—Tb^iv^	2.528 (3)
Tb—O3^ii^	2.528 (3)	O4—Tb^iv^	2.518 (3)
Tb—C7^ii^	2.878 (3)	C8—O5	1.241 (4)
N—C5	1.329 (4)	C8—O6	1.263 (4)
N—C1	1.340 (4)	O7—H7A	0.8302
C1—C2	1.392 (5)	O7—H7B	0.8454
C1—C6	1.506 (5)	O8—H8A	0.8470
C2—C3	1.387 (6)	O8—H8B	0.8095
C2—H2A	0.9293	O9—H9A	0.8057
C3—C4	1.400 (5)	O9—H9B	0.8018
C3—C7	1.500 (5)	O10—H10A	0.8390
C4—C5	1.396 (5)	O10—H10B	0.8498
C4—H4A	0.9271		
			
O2^i^—Tb—O6	149.38 (9)	C5—N—C1	119.3 (3)
O2^i^—Tb—O8	97.26 (11)	C5—N—Tb	120.1 (2)
O6—Tb—O8	73.97 (11)	C1—N—Tb	120.3 (2)
O2^i^—Tb—O1	75.64 (9)	N—C1—C2	122.2 (3)
O6—Tb—O1	128.99 (8)	N—C1—C6	114.5 (3)
O8—Tb—O1	141.98 (10)	C2—C1—C6	123.2 (3)
O2^i^—Tb—O9	75.06 (9)	C3—C2—C1	118.5 (3)
O6—Tb—O9	94.10 (10)	C3—C2—H2A	120.6
O8—Tb—O9	142.78 (10)	C1—C2—H2A	120.9
O1—Tb—O9	72.40 (10)	C2—C3—C4	119.5 (3)
O2^i^—Tb—O7	75.90 (9)	C2—C3—C7	122.3 (3)
O6—Tb—O7	73.49 (10)	C4—C3—C7	118.2 (3)
O8—Tb—O7	71.63 (10)	C5—C4—C3	117.6 (3)
O1—Tb—O7	138.26 (9)	C5—C4—H4A	121.2
O9—Tb—O7	71.18 (9)	C3—C4—H4A	121.2
O2^i^—Tb—N	133.27 (9)	N—C5—C4	122.7 (3)
O6—Tb—N	64.57 (9)	N—C5—C8	113.9 (3)
O8—Tb—N	129.16 (11)	C4—C5—C8	123.3 (3)
O1—Tb—N	64.56 (9)	O2—C6—O1	124.9 (3)
O9—Tb—N	70.47 (9)	O2—C6—C1	118.5 (3)
O7—Tb—N	119.42 (10)	O1—C6—C1	116.6 (3)
O2^i^—Tb—O4^ii^	125.16 (9)	C6—O1—Tb	123.2 (2)
O6—Tb—O4^ii^	82.13 (10)	C6—O2—Tb^iii^	135.5 (2)
O8—Tb—O4^ii^	76.72 (10)	O4—C7—O3	122.2 (3)
O1—Tb—O4^ii^	77.54 (9)	O4—C7—C3	120.2 (3)
O9—Tb—O4^ii^	137.60 (9)	O3—C7—C3	117.6 (3)
O7—Tb—O4^ii^	144.19 (9)	O4—C7—Tb^iv^	60.86 (18)
N—Tb—O4^ii^	69.89 (9)	O3—C7—Tb^iv^	61.32 (18)
O2^i^—Tb—O3^ii^	74.43 (9)	C3—C7—Tb^iv^	177.3 (2)
O6—Tb—O3^ii^	126.72 (10)	C7—O3—Tb^iv^	92.6 (2)
O8—Tb—O3^ii^	70.88 (10)	C7—O4—Tb^iv^	93.4 (2)
O1—Tb—O3^ii^	71.25 (9)	O5—C8—O6	126.4 (3)
O9—Tb—O3^ii^	137.04 (9)	O5—C8—C5	118.0 (3)
O7—Tb—O3^ii^	127.92 (9)	O6—C8—C5	115.6 (3)
N—Tb—O3^ii^	112.14 (9)	C8—O6—Tb	125.7 (2)
O4^ii^—Tb—O3^ii^	51.80 (9)	Tb—O7—H7A	129.7
O2^i^—Tb—C7^ii^	100.00 (10)	Tb—O7—H7B	101.9
O6—Tb—C7^ii^	104.70 (10)	H7A—O7—H7B	128.4
O8—Tb—C7^ii^	71.93 (10)	Tb—O8—H8A	125.8
O1—Tb—C7^ii^	72.68 (9)	Tb—O8—H8B	127.7
O9—Tb—C7^ii^	144.85 (10)	H8A—O8—H8B	105.4
O7—Tb—C7^ii^	142.43 (9)	Tb—O9—H9A	96.4
N—Tb—C7^ii^	90.95 (10)	Tb—O9—H9B	140.3
O4^ii^—Tb—C7^ii^	25.75 (10)	H9A—O9—H9B	122.1
O3^ii^—Tb—C7^ii^	26.05 (9)	H10A—O10—H10B	95.6

Symmetry codes: (i) −*x*+1, *y*+1/2, −*z*+3/2; (ii) *x*, −*y*+3/2, *z*−1/2; (iii) −*x*+1, *y*−1/2, −*z*+3/2; (iv) *x*, −*y*+3/2, *z*+1/2.

### Hydrogen-bond geometry (Å, º)

**Table:**

*D*—H···*A*	*D*—H	H···*A*	*D*···*A*	*D*—H···*A*
O7—H7*A*···O5^v^	0.83	1.98	2.642 (4)	136
O7—H7*B*···O4^vi^	0.84	2.45	2.966 (4)	120
O8—H8*A*···O5^v^	0.85	2.04	2.822 (4)	152
O8—H8*B*···O10^ii^	0.81	1.95	2.760 (5)	174
O9—H9*A*···O1^i^	0.81	2.00	2.808 (4)	179
O9—H9*B*···O3^vii^	0.81	2.00	2.805 (4)	179
O10—H10*A*···O6^viii^	0.84	2.33	2.977 (5)	134
O10—H10*B*···O7^viii^	0.85	2.55	3.169 (5)	130

Symmetry codes: (i) −*x*+1, *y*+1/2, −*z*+3/2; (ii) *x*, −*y*+3/2, *z*−1/2; (v) −*x*, *y*+1/2, −*z*+3/2; (vi) *x*, −*y*+5/2, *z*−1/2; (vii) *x*, *y*+1, *z*; (viii) *x*, *y*−1, *z*.
